# Relationship between tracheobronchoscopic score and bronchoalveolar lavage red blood cell numbers in the diagnosis of exercise‐induced pulmonary hemorrhage in horses

**DOI:** 10.1111/jvim.15676

**Published:** 2019-12-27

**Authors:** Carolina M. Lopez Sanchez, Clark Kogan, Jenifer R. Gold, Debra C. Sellon, Warwick M. Bayly

**Affiliations:** ^1^ Department of Veterinary Clinical Sciences Washington State University Pullman Washington; ^2^ Center for Interdisciplinary Statistical Education and Research Washington State University Pullman Washington

**Keywords:** barrel racing, EIPH, endoscopy, equine, exercise

## Abstract

**Background:**

Exercise‐induced pulmonary hemorrhage (EIPH) is diagnosed and its severity assessed by post‐exercise tracheobronchoscopy, and enumeration of bronchoalveolar lavage fluid red blood cells (BALFRBC). Minimal information is available regarding the relationship of tracheobronchoscopy score to BALFRBC number.

**Objective:**

Evaluate the relationship between BALFRBC number and tracheobronchoscopy scores and determine their diagnostic sensitivities.

**Animals:**

Nine sedentary horses, 21 fit Thoroughbreds, 129 Barrel Racers.

**Methods:**

Normal BALFRBC number and the effect of bronchoalveolar lavage (BAL) on it were evaluated by performing 2 BALs 24 hours apart in sedentary horses. Tracheobronchoscopy followed by BAL was performed 247 times on 150 horses after treadmill, racetrack, or barrel racing exercise. Lastly, a BALFRBC diagnostic threshold number that optimized the geometric mean of the sensitivity and precision (F1‐score) was determined using Bayesian analysis.

**Results:**

No increase in BALFRBC occurred after the second BAL (mean ± SD, 304 ± 173/μL). Tracheobronchoscopy scores ranged from 0 (n = 112) to 4 (n = 4) and BALFRBC ranged from 102 to 4605268/μL. Spearman correlation between tracheobronchoscopy score and BALFRBC was weak (*P* < .001; rs = 0.42) with large ranges of BALFRBC associated with each tracheobronchoscopy score. The highest F1‐score occurred for a BALFRBC threshold number = 992/μL. Seventy‐five tracheobronchoscopy scores equaled 0 although BALFRBC number was ≥992/μL. Sensitivity of tracheobronchoscopy for diagnosing EIPH was poor (0.59; 95% confidence intervals [CI], 0.49‐0.68), compared to BALFRBC number ≥992/μL (0.93; 95% CI, 0.88‐0.96).

**Conclusions and Clinical Importance:**

False negatives are common with tracheobronchoscopy. Follow‐up determination of BALFRBC may be indicated for tracheobronchoscopy scores = 0 before EIPH can be ruled out.

**Abbreviations**
BAL: bronchoalveolar lavage
BALFRBC: bronchoalveolar lavage fluid red blood cell number
EIPH: exercise‐induced pulmonary hemorrhage
PBS: phosphate‐buffered saline
RBC: red blood cells
ROC: receiver operating characteristic
TBE: tracheobronchoscopic examination

## 1 INTRODUCTION

Exercise‐induced pulmonary hemorrhage (EIPH) is defined as the presence of blood within the lower airways and is commonly seen in horses after intense exercise.^1^ The diagnosis of EIPH relies on the observation of blood in the upper airway, trachea, or large bronchi during tracheobronchoscopic examination (TBE), counting red blood cells (RBC) in the fluid collected from a post‐exercise bronchoalveolar lavage (BAL), or both.^2^

Although TBE assigns 1 of 5 possible scores (0‐4) as a reflection of EIPH severity, evaluation by BAL is semi‐quantitative and has been used to assess the severity of EIPH in horses, cyclists, and greyhounds.^2^, ^3^, ^4^ Distal segments of a lung are lavaged after exercise, as much lavage fluid as possible recovered, and the number of RBC in that fluid (BALFRBC) determined. The diagnostic validity of BAL with respect to EIPH has been questioned because of the possibility that the procedure itself might induce hemorrhage from the airway mucosa and iatrogenically increase the post‐exercise BALFRBC number.^5^ However, we could find little evidence that this concern has been investigated in horses.^6^

Bronchoalveolar lavage is not commonly performed after TBE in the short‐term post‐exercise period for the diagnosis of EIPH, and we could find no criteria regarding the BALFRBC number that is considered positive for the condition. Some studies have utilized post‐treadmill exercise BALFRBC number to assess the effects of putative prophylactic treatments for EIPH, but none of them designated horses as positive or negative for EIPH on the basis of a given number.^7^, ^8^, ^9^, ^10^

Tracheobronchoscopic examination is easier to perform than BAL and consequently is the diagnostic method of choice for EIPH. Criteria for assigning a particular TBE EIPH score severity have been described in detail.^11^ The TBE EIPH scores are assigned according to observations made 30 minutes to 2 hours after exercise, with a score of 0 signifying no visible evidence of EIPH. Although the specificity of TBE is apparently very high (low rate of false positives), it is also assumed to have a high sensitivity. However, this assumption has not been justified and the sensitivity of TBE may well be lower than presumed, because the lack of visible TBE evidence of EIPH does not indicate that hemorrhage did not occur.

Our overall objective was to evaluate the relationship between BALFRBC and TBE EIPH scores after strenuous exercise in horses. First, because of uncertainty as to whether the BALFRBC number can be affected by the BAL procedure itself, we showed that the BAL procedure did not influence BALFRBC number. We then investigated the relationship between BALFRBC and TBE EIPH scores by correlating EIPH scores with BALFRBC numbers in a large population of horses after treadmill and racetrack exercise, and barrel racing. Lastly, we identified a BALFRBC number that could serve as a threshold value for purposes of diagnosing a horse as positive or negative for EIPH and used this number to determine and compare the sensitivities and specificities of TBE score and BALFRBC as diagnostic tests of EIPH after supramaximal exercise.

## 2 MATERIALS AND METHODS

### 2.1 Effects of the BAL procedure on lavage RBC number

Nine healthy sedentary adult horses (5 Thoroughbreds, 3 Arabians, 1 Quarter Horse; aged 9‐15 years) that had not exercised for at least 3 months were used. Two BALs were performed on each horse 24 hours apart by blindly passing a flexible balloon‐tipped catheter into the right or left bronchial tree, as described below.

### 2.2 Determining the correlation between TBE score and BALFRBC number

To determine the correlation between TBE score and BALFRBC number, 247 TBE, followed by BAL, were performed 45‐90 minutes after strenuous exercise on 150 different Thoroughbreds (mean ± SD age, 7 ± 1.8; range, 4‐13 years; body weight, 495 ± 38 kg), Quarter Horses, and Appendix Quarter Horses (8 ± 11.5; range, 3‐23 years) for both breeds. Exercise modalities consisted of supramaximal treadmill exercise (20 Thoroughbreds, 75 runs) and breezing and simulated racing on a racetrack (7 of the 20 horses that exercised on the treadmill plus 1 other; 27 runs) and barrel racing (129 Quarter Horses and Appendix horses, 145 runs).

### 2.3 Supramaximal treadmill exercise

Each horse galloped to fatigue at the speed for which the calculated oxygen requirement was 115% of its maximum oxygen consumption ($\dot{V}$O_2_max). This speed was calculated from the regression equation for the linear section of each animal's $\dot{V}$O_2_‐speed relationship that was generated after completion of an incremental treadmill test to measure $\dot{V}$O_2_max^12^, ^13^, ^14^ 4‐6 days before the supramaximal test. For the supramaximal test, the horses warmed up by trotting for 3 minutes at 40% $\dot{V}$O_2_max, after which the treadmill was accelerated to the predetermined 115% $\dot{V}$O_2_max speed. Each horse galloped at this speed until it could no longer keep pace with the treadmill.

### 2.4 Racetrack exercise

Racetrack exercise took place on the Washington State University Hitchcock Research Racetrack in the form of 800 m breezes and 1600 m simulated races, and Emerald Downs Racetrack, Auburn, Washington, where 2 1100 m simulated races were completed from the starting gate. A minimum of 7 days elapsed between each test on the Hitchcock Research Racetrack and 13 days between the 2 1100 m simulated races.

### 2.5 Barrel racing

Horses were examined with the permission of event organizers and owners at events in Washington, Idaho, Oregon, and Montana. The events typically were 2‐4 days long with a variety of competitive levels. A minimum of 6 days elapsed between events.

### 2.6 Tracheobronchoscopic examination and EIPH scoring

Tracheobronchoscopy was performed 45‐60 minutes post‐exercise in the Thoroughbreds and 45‐90 minutes after completing barrel races in the other horses. Before the TBE, all horses were sedated with xylazine (0.5‐1.0 mg/kg IV; MWI Animal Health, Boise, ID) and acepromazine maleate (0.02‐0.6 mg/kg IV; Boehringer Ingelheim Vetmedica, Inc, St. Joseph, Missouri). A 300‐cm long, 8‐mm diameter flexible fiber‐optic endoscope was passed via a ventral nasal meatus into the trachea and down to the level of the carina for the Thoroughbreds, and into the right or left mainstem bronchus or both depending upon whether blood was noted in the right, left, both, or neither bronchus for the barrel racers. The presence and severity of EIPH was graded according to a scale defined by the quantity of blood observed in the airways.^11^

### 2.7 Bronchoalveolar lavage

Bronchoalveolar lavage was performed immediately after TBE, except for the initial study using sedentary horses, which did not entail TBE. For the Thoroughbreds, it consisted of blind instillation of 300 mL of phosphate‐buffered saline (PBS) into 1 lung, through a flexible balloon‐tipped catheter (Bivona Medical Technologies, Gary, Indiana; 102 samples). With the barrel racers, a catheter was passed down the biopsy channel of the endoscope at the conclusion of TBE and 300 mL PBS infused through it. After infusion of the fluid, it was aspirated until a detectable increase in resistance to its collection occurred. The RBC in the BALF were manually counted under ×40 microscope power using a hemocytometer. The number of erythrocytes in the BALF was expressed as RBC/μL.

### 2.8 Statistical analysis

The correlation between EIPH TBE score and post‐exercise BALFRBC number was calculated using a Spearman rank correlation with data collected after barrel racing, treadmill, and racetrack exercise events. The effect of prior BALF evaluation on BALFRBC number was assessed using a 1‐way repeated‐measures analysis of variance. In all instances, significance was set at *P* < .05.

Sensitivity and specificity of TBE in exercised horses along with the population characteristics of BALFRBC numbers in both exercised and non‐exercised Thoroughbreds were estimated using a Bayesian analysis similar to that described previously.^15^ Data collected after the 102 supramaximal treadmill or racetrack exercise tests were used for Bayesian analysis of exercised horses because of the tight control on time post‐exercise when TBE and BAL were performed (45‐60 minutes). The BALFRBC numbers determined from the 2 BALs performed 24 hours apart on the 9 sedentary horses were used as the best source of true EIPH negative results. The logarithm of the BALFRBC number was modeled using a normal distribution with separate means and SDs for exercised and non‐exercised horses. Uninformative normal priors were specified on the means of both normal distributions (mean, 0; SD, 100), and uninformative uniform (range, 0‐100) priors were specified for the SDs of both normal distributions. The TBE positive and negative outcomes were modeled as Bernoulli random variables with probability of a positive test result conditional on EIPH. An uninformative uniform 0‐1 prior was used for the probability of a positive TBE conditional on EIPH to reflect our lack of knowledge of the sensitivity of this test. An informative uniform prior (range, 0‐0.01) was used for the probability of a positive TBE conditional on no EIPH to reflect our understanding that a positive TBE cannot occur without EIPH. Probability of EIPH was fixed at 0 for non‐exercised horses and was modeled as Bernoulli with uninformative uniform (range, 0‐1) prior probability. The BALFRBC distributional parameters were used to estimate a diagnostic threshold, striking a balance between the costs of false positives and false negatives with the geometric mean of the sensitivity and precision, which is called the F1‐score.^16^

This Bayesian model was run for 15 000 iterations for each of 4 chains, and the first 5000 “burn‐in” samples were discarded. A sensitivity analysis was conducted to assess the sensitivity of the mean of the posterior distributions to small changes in the prior of the TBE false‐positive rate. In addition to the uniform prior with range 0‐0.01, uniform priors were tested at ±10% for the upper range (eg, 0‐0.009 and 0‐0.011) to assess any effects on the posterior means. A receiver operating characteristic (ROC) curve was included to show error rates for the range of potential BALFRBC threshold numbers. Maximum a posteriori estimates of BALFRBC normal distribution parameters were used to obtain an ROC curve for BALFRBC numbers. From the ROC curve, the optimal F1‐score threshold was determined for the BALFRBC number.

A Bayesian analysis was conducted to estimate log BALFRBC mean and variance for true positive and true negative horses as well as posterior distributions on sensitivity and specificity for TBE.

The sensitivity (true positive rate) and specificity (true negative rate) were reported conditional on the optimal F1‐score BALFRBC threshold. All statistical analyses were performed using R (R Core Team. R: A language and environment for statistical computing. R Foundation for Statistical Computing, Vienna, Austria, https://www.r-project.org/) and JAGS 4.3.0.^17^

## 3 RESULTS

### 3.1 BALFRBC in sedentary horses

The BALF recovered from these horses was very clear and colorless. Bronchoalveolar lavage using the soft balloon‐tipped tube on the sedentary horses had no effect on the BALFRBC number after a second BAL 24 hours after the first procedure (*P* = .47). All BALFRBC numbers recovered from both BALs in these horses were < 700 RBC/μL, and the difference between the first and second BALFRBC numbers ranged from an increase of 248 RBC/μL to a decrease of 107 RBC/μL. The mean ± SD BALFRBC number for the first BAL was 286 ± 160/μL (range, 75‐617), while that for the second procedure was 322 ± 194/μL (range, 25‐532). No evidence of erythrophagocytosis was observed in any of the collected BALF.

### 3.2 Relationship between TBE EIPH score and BALFRBC number

**Figure 1:**
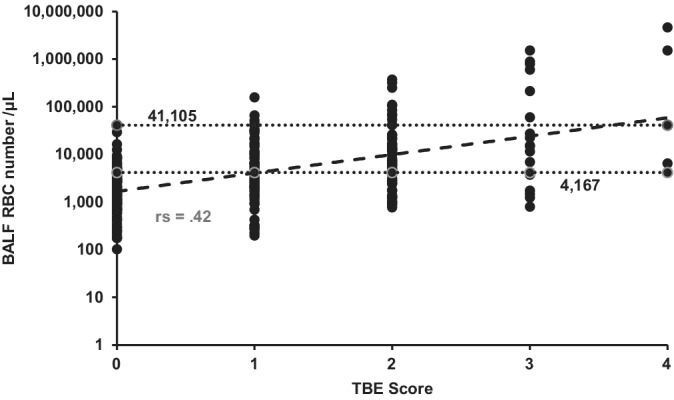
Correlation (rs = 0.42) between tracheobronchoscopic examination (TBE) score and post‐exercise bronchoalveolar lavage fluid red blood cell number, using log10 scaling, based on results from 247 treadmill, race track, and barrel racing events. The regression line is reflected by the dashed line. Bronchoalveolar lavage fluid red blood cell numbers from 4176 to 41 105/μL were associated with all TBE exercise‐induced pulmonary hemorrhage scores as indicated by the dotted lines

**Figure 2:**
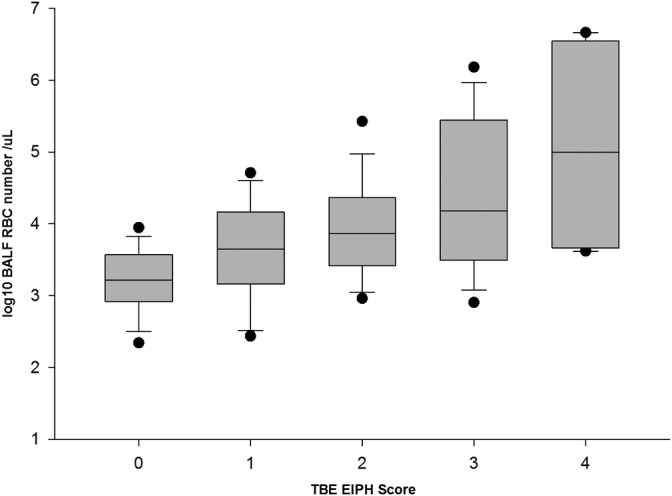
Median interquartile range (IQR) number of the log10 bronchoalveolar lavage fluid red blood cell number for each tracheobronchoscopic examination score from 0 to 4, where the box represents the IQR, the whiskers represents the 5th/95th percentile and the dots show the minimum and maximum observations

The Spearman rank correlation between TBE score and BALFRBC number was significant but weak (*P* < .001; r_s_ = 0.42; Figures 1 and 2). Numbers of BALFRBC between 4167 and 41 105/μL were associated with TBE EIPH scores from 0 to 4 (Table 1, Figure 1). The color of the BALF also varied considerably, ranging from a colorless to dark red when TBE score = 0, and from pink to dark red for TBE scores of 1‐4.

**Table 1:** Mean ± standard deviation, median, and range of bronchoalveolar lavage fluid red blood cell **(**BALFRBC) numbers associated with each tracheobronchoendoscopy (TBE) exercise‐induced pulmonary hemorrhage score from 247 exercise events

TBE score	n	BALFRBC number		
		Mean (±SD)/μL	Median/μL	Range/μL
0	112	3227 ± 5146	1641	102‐41 105
1	59	13 082 ± 23 925	4470	199‐158 513
2	54	34 177 ± 73 350	7350	775‐371 530
3	18	232 742 ± 419 737	15 162	801‐1 520 122
4	4	1 534 009 ± 1 877 974	763 251	4176‐4 605 268

### 3.3 Sensitivity and specificity of TBE score and BALFRBC number

Based on TBE, the prevalence of EIPH in the barrel racers was 52%, whereas that in the Thoroughbreds was 59%. The optimal F1‐score cutoff threshold for BALFRBC was 992/μL, where BALFRBC number ≥992/μL was indicative of EIPH. The prevalence of EIPH using BALFRBC number > 992/μL 45‐90 minutes after exercise was 96% for Thoroughbreds and 66% for barrel racers. Of the 102 pairs of data from the Thoroughbreds, there was 1 instance (0.9%) of a TBE score ≥ 1 but BALFRBC number ≤992/μL (ie, a BALF false negative) and 39 samples that were positive for EIPH based on BALF analysis but negative on TBE (ie, EIPH score = 0; Table 2). The TBE sensitivity was estimated at 0.59 (95% credible interval, 0.49‐0.68; Table 3). The BALFRBC estimated sensitivity at our determined threshold was 0.93 (95% credible interval, 0.88‐0.96), and the estimated specificity was 0.95 (95% credible interval, 0.73‐1.0; Table 3).

**Table 2:** Tracheobronchoendoscopy (TBE) exercise‐induced pulmonary hemorrhage scores and bronchoalveolar lavage fluid red blood cell (BALFRBC) numbers from 102 Thoroughbred exercise events on a treadmill or racetrack used in the Bayesian analysis

	BALFRBC number+	BALFRBC number−	Total
TBE score+	59	1	60
TBE score−	39	3	42
Total	98	4	102

A positive EIPH result was assumed for TBE score ≥1 and/or BALFRBC number ≥992/μL, and a negative EIPH result was associated with a TBE score = 0 and BALFRBC number < 992/μL.

**Table 3:** Sensitivities and specificities of tracheobronchoscopic endoscopy (TBE) and BAL as diagnostic tests for EIPH, based on Bayesian analysis and using a BALFRBC number ≥ 992/μL or TBE ≥1 as positive for EIPH, and a TBE score = 0 and BALFRBC number < 992/μL as negative for EIPH

	TBE	95% CI	BALFRBC number	95% CI
Sensitivity	0.59	0.49‐0.68	0.93	0.88‐0.96
Specificity	0.995	0.99‐1	0.95	0.73‐1

Abbreviations: BAL, bronchoalveolar lavage fluid; BALFRBC, bronchoalveolar lavage fluid red blood cell; CI, confidence interval; EIPH, exercise‐induced pulmonary hemorrhage; TBE, tracheobronchoscopic examination.

## 4 DISCUSSION

The reported prevalence of EIPH based on TBE is highest in racing Thoroughbreds, with other strenuous athletic activities having lower rates of occurrence, including observed prevalences of 45%^18^ and 54%^10^ in barrel racers. This finding confirms the high prevalence of EIPH in horses after strenuous exercise of different types.^10^, ^18^, ^19^, ^20^, ^21^, ^22^ However, considerable disparity was observed between the diagnostic sensitivity of TBE as a diagnostic test for EIPH when compared to BALFRBC number.

No previous studies have aimed to address the correlation between TBE score and BALFRBC number and their respective sensitivities and specificities as diagnostic tests for EIPH. A statistically significant relationship was found between EIPH TBE score and BALFRBC number. However, the correlation between these 2 diagnostic methods was weak. The SD of the BALFRBC number for each TBE EIPH score was higher than the corresponding mean, and the range of RBC numbers associated with each score was very large (Figure 1). We clearly demonstrated that a horse cannot be deemed EIPH‐negative based on failure to visualize EIPH with TBE. The lack of visual evidence of EIPH should not be equated with the absence of EIPH, because such horses still can have a strongly positive BALFRBC number. In some instances, there also was marked visual evidence of EIPH based on the obviously pink or red color of the recovered BALF from horses with a TBE score of 0. This was in contrast to the lack of color to the BALF collected from the sedentary horses.

It has been unclear as to whether the BAL procedure itself might be associated with airway hemorrhage. One previous report documented effects of repeated weekly BAL procedures for 10 weeks in 4 non‐exercised horses and found that there was no change in BALFRBC number over the course of the study.^23^ However, because BALFRBC numbers decreased rapidly in the first 3 days after instillation of 40 mL of autologous blood into lung segments,^24^ we felt it was important to further assess whether the BAL procedure itself could induce hemorrhage and inadvertently increase post‐exercise BALFRBC by evaluating the effects on this variable of performing 2 BALs in a period of 24 hours. The results indicated that performing the first BAL had no effect on the RBC number recovered in the subsequent procedure. Furthermore, the mean ± SD for each BAL was very similar to that previously reported in non‐exercised horses lavaged weekly for 10 weeks (309 ± 73/μL).^23^ These numbers were lower than those previously reported for non‐exercised horses (844 ± 123/μL).^6^ However, those horses had been exercised regularly until 4‐6 weeks before 2 BALs were performed 1 week apart. Despite the fact that BALFRBC numbers decrease markedly soon after instillation of known volumes of blood, erythrocytes can be detected 2 and 3 weeks, respectively, after a single bout of strenuous exercise,^24^, ^25^, ^26^ which is why we selected horses that had not been exercised for at least 3 months before this portion of the investigation. The highest BALFRBC number recorded from the 18 BALs performed on the sedentary horses was 617/μL, and the subsequently calculated EIPH positive RBC threshold number (992/μL) was > 3 SDs higher than the mean BALFRBC numbers in these horses. Consequently, it seems very unlikely that the BAL procedure per se induces airway hemorrhage provided that an appropriate technique is utilized. It is unclear why a small number of RBCs were present in the BALF of sedentary horses, but this finding is not without precedent.^6^ It is unlikely that such findings reflect previous bouts of EIPH because of the lengthy period between any previous exercise and BAL and the lack of evidence of erythrophagocytosis. Some diapedesis of blood into the smallest airways may be normal, because some leucocytes also are normally found in the airways of healthy horses.^6^, ^27^

The sensitivity of a diagnostic test is a measure of its accuracy in detecting cases that are truly positive for the condition in question, whereas the test's specificity refers to the likelihood that it will correctly identify cases that do not have the condition (true negatives). With respect to EIPH, the clinical emphasis is on correctly identifying all horses that have experienced pulmonary hemorrhage, especially those in which the occurrence is severe enough to be performance limiting. Anecdotally, the sensitivity of TBE has been presumed to be high, and management and therapeutic decisions often are based on whether or not a horse is deemed to be EIPH‐positive on TBE. Others have suggested that BAL is a more sensitive test than TBE on the basis of EIPH prevalence calculations derived from BAL findings in actively exercising racehorses.^8^, ^28^ Although the diagnosis of EIPH was based on the detection of hemosiderin granules and hemosiderophages as well as erythrocytes in those studies, the evidence indicated that the prevalence of EIPH was >90%, which was well above reported prevalences of up to 80% based on a single TBE in other groups of racehorses.^29^, ^30^ Bronchoalveolar lavage was postulated to be a more sensitive diagnostic test because of these differences in prevalence, because sensitivity was not actually determined. We were unable to find any published calculations of the sensitivity or specificity of TBE or BALFRBC number, although reference also has been made to the possibility that TBE can be associated with false‐negative results.^11^

Calculation of a diagnostic test's sensitivity and specificity often is based on the existence of a gold standard upon which classification of a test result as truly positive, truly negative, falsely positive, or falsely negative is based. Because we were not confident that the sensitivity of the TBE test was high enough to consider it a gold standard, we could not determine the sensitivity and specificity of BALF in the classical way. To remedy this conundrum, we employed a Bayesian analysis to utilize our prior understanding of the specificity of the TBE, and data collected from the 102 tests on Thoroughbreds was used to estimate the sensitivity and specificity for both TBE and BALFRBC number. Computation of the sensitivity and specificity require that a clear discretization be made from test results (ie, a clear threshold is specified for assuming EIPH has occurred). Although several studies have empirically concluded that measuring post‐exercise BALFRBC number is a more sensitive test,^6^, ^7^, ^27^, ^28^ none of them has provided a numerical estimate of sensitivity of TBE or BAL, or suggested a threshold upon which a determination that a horse is either EIPH‐positive or EIPH‐negative can be based. As the results of the study of the sedentary horses showed, the presence of some erythrocytes in BALF is normal. Consequently, a diagnosis of EIPH cannot be solely based on observing some RBC in BALF. To avoid making an arbitrary designation for a BALF threshold, we elected to choose a threshold which maximized the F1‐score. Because the aim of these calculations was to determine a diagnostic EIPH threshold for BALFRBC number that could be applied to any horse that had undergone high‐intensity exercise of any type in the absence of known information other than a negligible rate of false positives, the Bayesian model also assumed uninformative prior knowledge regarding exercise and EIPH history and that the likelihood of EIPH positives and negatives was balanced (ie, 50% for each).

The sensitivity and specificity for TBE and model parameters that characterized the distribution of BALFRBC numbers in both exercised and non‐exercised Thoroughbreds were estimated. We used our estimated BALFRBC number distributions to determine an optimal BALFRBC threshold that maximized the F1‐score. The F1‐score is a measure of a test's accuracy and is computed as the harmonic mean of the precision and the recall of the test. The BALFRBC number that corresponded to the highest F1‐score was 992/μL. This number therefore was designated as the recommended EIPH threshold number from which calculations of sensitivity and specificity for BALFRBC number were subsequently derived.

Tracheobronchoscopic examination is easier to perform than BAL and consequently is the diagnostic method of choice for most veterinarians. The specificity and sensitivity of TBE score were 0.99 and 0.59, respectively. The high specificity is compatible with the fact that it is almost impossible to have a false‐positive result on TBE after strenuous exercise. Any evidence of pulmonary hemorrhage observed is almost certainly associated with EIPH. Conversely, the low sensitivity for TBE was a result of many horses with negative TBE having BALFRBC numbers that were much higher than were observed in the non‐exercised horses. This and the weak correlation between TBE score and BALFRBC number suggest that, before ruling out a diagnosis of EIPH, both TBE and BAL should be performed if the TBE score = 0, because the latter is only a measure of visible hemorrhage and the possibility that blood is present in the airways beyond the visual field of TBE cannot be excluded. In the event of a TBE score > 0, a BAL is not indicated because of the negligible number of false positives linked to TBE.

It is unclear whether the time that elapsed from the completion of exercise until TBE was conducted influenced TBE score and TBE sensitivity. Because variations in this duration affect the results of TBE,^31^ we sought to keep this time as consistent as possible in the portion of the study that used Thoroughbreds. The rate at which blood moves from the distal airways to the trachea appears to vary within and between individual horses because of factors such as the volume of hemorrhage and the presence or absence of post‐exercise coughing. Therefore, in horses that have performed strenuous exercise poorly and it is not possible to follow a negative TBE (score = 0) with BAL, repetition of the TBE 30‐60 minutes later may be warranted before ruling out the diagnosis of EIPH.

A single unexpected instance occurred in which the TBE EIPH score was >0 but the BALFRBC number was <992/μL. This instance was regarded as a false‐negative BALFRBC result. The threshold RBC was determined from the Bayesian statistical analysis rather than direct scientific evaluation and was therefore the best possible estimate based on the available data, so the occurrence of a false‐negative BAL result was not surprising. The finding also could have been an indication that all or most of the hemorrhage into the airways had cleared the smaller airways by the time the BAL was performed or that the region of lung that was lavaged was not representative of the area from which the EIPH emanated in that particular horse.

In conclusion, the diagnosis of EIPH cannot always be based on TBE. The relationship between BAL and TBE is weak to moderate at best, and it is important that veterinarians, horse owners, and trainers be aware of this poor correlation. Practitioners might be advised to perform BAL on horses that have not performed well but have a TBE score = 0, before completely ruling out EIPH. If the goal is to identify whether a horse is positive for EIPH, then BALFRBC number is the single test of choice because it has a much higher sensitivity (0.93 versus 0.59). However, it is more practical to first perform TBE because it is easier to do. If the TBE = 0, then a BAL can be performed subsequently because of the high false‐negative rate with TBE.

## CONFLICT OF INTEREST DECLARATION

Dr. Bayly and Dr. Sellon are former members of the Board of Directors of the American Association of Equine Practitioners. Dr. Bayly is currently a member of the Grayson‐Jockey Club Research Advisory Committee, but was not at the time of funding. The other authors have no conflicts of interest.

## OFF‐LABEL ANTIMICROBIAL DECLARATION

Authors declare no off‐label use of antimicrobials.

## INSTITUTIONAL ANIMAL CARE AND USE COMMITTEE (IACUC) OR OTHER APPROVAL DECLARATION

Approved by the Washington State University IACUC.

## HUMAN ETHICS APPROVAL DECLARATION

Authors declare human ethics approval was not needed for this study.
